# Preoperative computed tomography-guided transscapular sens-cure needle localization for pulmonary nodule located behind the scapula

**DOI:** 10.1186/s13019-023-02304-3

**Published:** 2023-07-05

**Authors:** Zi-Wei Lu, Yue-Yue Liu, Yong-Gang Li, Lu-Lu Lv

**Affiliations:** 1 Department of Radiology, The First Affiliated Hospital of Soochow University, Suzhou, China; 2 Department of Radiology, Xuzhou Central Hospital, Xuzhou, China; 3 Institute of Medical Imaging, Soochow University, Suzhou, China; 4 National Clinical Research Center for Hematologic Diseases, The First Affiliated Hospital of Soochow University, Suzhou, China; 5 Suzhou Key Laboratory of Intelligent Medicine and Equipment, Suzhou, China

**Keywords:** Computed tomography, Sens-cure needle, Scapula, Pulmonary nodule

## Abstract

**Background:**

Video-assisted thoracoscopic surgery (VATS) is an approach that is commonly used to resect pulmonary nodules (PNs). However, when these PNs are located behind the scapula, a transscapular access approach is generally required. In this study, the safety, efficacy, and feasibility of preoperative computed tomography (CT)-guided Sens-cure needle (SCN) localization was assessed for PNs located behind the scapula.

**Methods:**

From January 2020 - June 2022, a total of 122 PN patients in our hospital underwent preoperative CT-guided SCN localization and subsequent VATS resection, of whom 12 (9.8%) exhibited PNs behind the scapula necessitating a transscapular approach for this localization procedure.

**Results:**

This study included 12 patients, each of whom had one PN located behind the scapula. The CT-guided transscapular SCN localization approach was successful in all patients, and no complications near the operative site were observed. The median localization time was 12 min, and 2 (16.7%) and 1 (8.3%) patients respectively developed pneumothorax and pulmonary hemorrhage after the localization procedure was complete. Wedge resection procedures for these PNs achieved technical success in all cases. Four patients were diagnosed with invasive adenocarcinomas and subsequently accepted lobectomy and systematic lymph node dissection. The median VATS duration and the median blood loss was 80 min and 10 mL, respectively. In total, 3, 5, and 4 PNs were respectively diagnosed as benign, mini-invasive adenocarcinomas, and invasive adenocarcinomas.

**Conclusion:**

Preoperative CT-guided transscapular SCN localization represents a safe, straightforward, and effective means of localizing PNs present behind the scapula.

## Introduction

Video-assisted thoracoscopic surgery (VATS) is an approach that is frequently employed in the resection of pulmonary nodules (PNs) [1–5], and the success of VATS wedge resection procedures has been greatly improved by the use of preoperative computed tomography (CT)-guided localization strategies [1–5]. Materials utilized to facilitate localization include micro-coils, hook-wire, radiolabeling materials, and certain liquid-based localization materials [6, 7]. However, the localization of PNs that are situated behind bone structures is challenging irrespective of the localization materials employed. While a succenturiate localization approach is often selected for these lesions located behind bony structures, when target PNs are instead obscured by the scapula, a transscapular approach is often employed because the scapula is thin and readily punctured, while its large size can make succenturiate pathway selection challenging [8].

Hook-wire localization is the most common approach [7], with 30/46 studies in a prior meta-analysis of preoperative PN localization having utilized a hook-wire approach [7]. However, rates of complications are relatively high in patients undergoing hook-wire localization, with many experiencing dislodgement (0.4–19.4%), pneumothorax (7.5–56.2%), and parenchymal hemorrhage (10.3–25.8%) [9]. In an effort to mitigate this operative morbidity, researchers have developed Sens-cure needles (SCN) as an alternative localization material that can be used in patients harboring PNs [9, 10]. The feasibility of SCN-based localization for PNs located behind the scapula, however, remains unclear.

This study was thus designed to gauge the safety, feasibility, and efficacy of preoperative CT-guided SCN localization for PNs present behind the scapula.

## Methods

The Ethics Committee of The First Affiliated Hospital of Soochow University approved this retrospective study, and the requirement for patient consent was waived owing to the study design.

### Study design

From January 2020 - June 2022, 122 PN patients underwent preoperative CT-guided SCN localization prior to VATS resection in our hospital, of whom 12 (9.8%) presented with PNs located behind the scapula such that they underwent localization performed via a CT-guided transscapular approach.

Patients eligible for study inclusion were: (a) individuals with PNs located behind the scapula; (b) individuals with PNs ≥ 6 mm in diameter; (c) individuals with PNs exhibiting a moderate-to-high malignancy risk based on clinical and radiological findings [11].

Patients were excluded if: (a) PN localization could be performed without the use of a transscapular approach; (b) the PN-pleura distance was > 30 mm; or (c) patients exhibited VATS contraindications.

### Preoperative analyses

Chest CT scans were used for preoperative PN detection and for the measurement of PN diameter values and the PN-pleura distance. PNs were classified as solid, ground-glass nodules (GGNs), or mixed GGNs. A transscapular localization approach was selected when the shortest PN-pleura line crossed the scapula [12].

### SCN structure

**Fig. 1:**
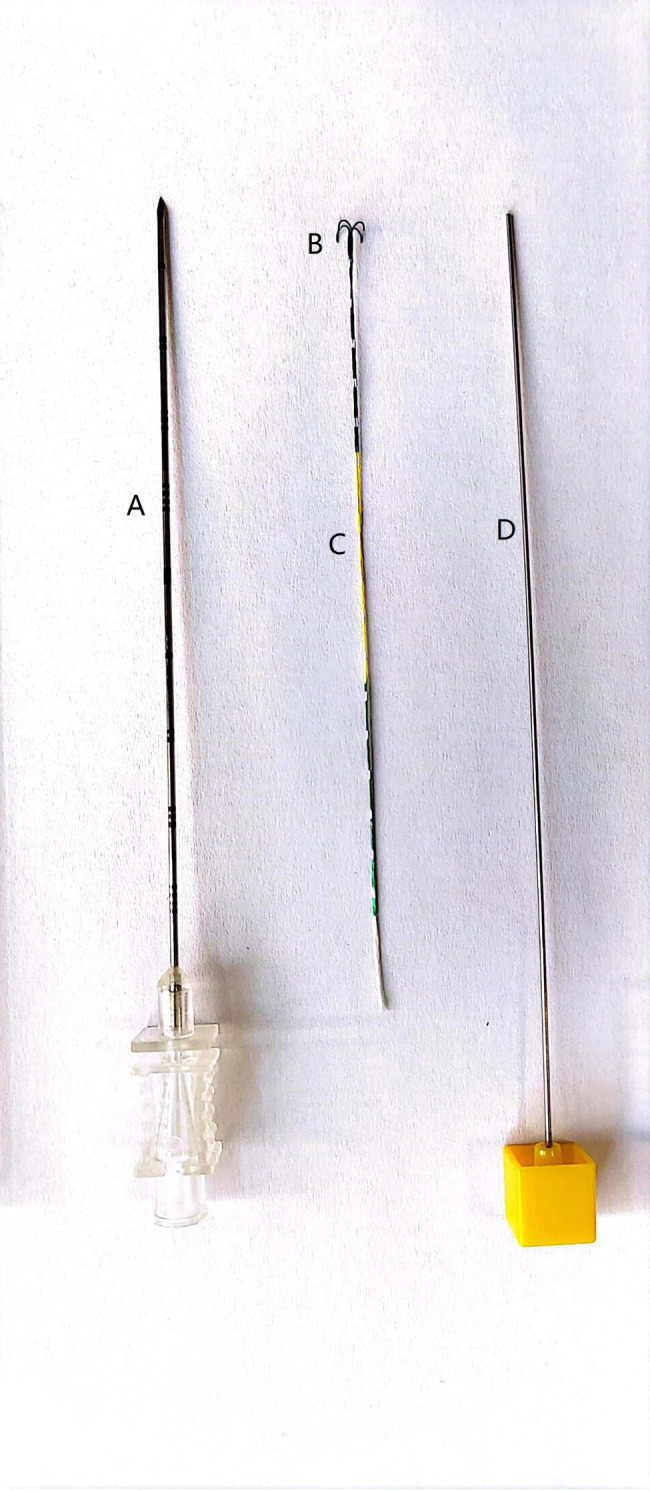
The parts of the SCN: **(a)** co-axial needle; **(b)** anchor claw; **(c)** suture; and **(d)** pusher

Individual SCNs (Ningbo SensCure Biotechnology Co., Ltd., Ningbo, China) were composed of four parts: (a) a co-axial needle; (b) an anchor claw; (c) a suture; and (d) a pusher (Fig. 1). These SCNs had a 19G diameter and were 100/150 mm in length.

### CT-guided scapular puncture

**Fig. 2:**
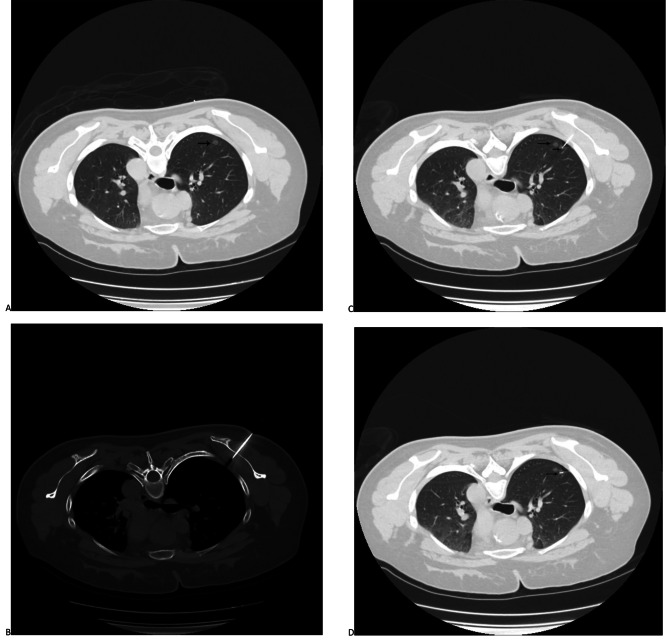
The procedures of the trans-scapula SCN localization. **(a)** Preoperative CT for the PN (arrow) behind the scapula; **(b)** The scapula was punctured; **(c)** The tip (short arrow) of the SCN was advanced near the PN (long arrow). **(d)** The anchor claw (arrow) of the SCN was released near the PN

A Somatom Semsation 64 CT instrument (SIEMENS, Forchheim, Germany) was used to conduct all procedures under local anesthetization. A thoracic CT scan was performed immediately before the procedure for each patient to establish the optimal needle pathway for the target PN (Fig. 2a). The scapula was then punctured by a 17G needle, which was advanced with rotation when the needle tip was in contact with the scapula. Repeated CT scanning was performed to ensure that the needle had not punctured the thoracic cavity and to assess needle tip localization (Fig. 2b).

### SCN localization protocols

Following the passage of the 17G needle through the scapula, a 19G SCN was used for pulmonary parenchymal puncture. Needle tip localization was confirmed through repeated CT scanning, and the needle position was adjusted as needed until the tip was ≤ 1 cm from the target PN (Fig. 2c). Following release buckle removal, the pusher was extended to the marked line. Following anchor claw release proximal to the PN (Fig. 2d), the pusher was removed prior to the retraction of the tip to a position located between the lung and the thoracic wall. The pusher was then reintroduced into the needle such that the tri-colored suture was forced from the needle, followed by the withdrawal of the needle and pusher. Procedure-associated complications were then detected through additional CT scanning.

### VATS procedure

VATS was conducted within 1–2 h after localization when possible. Briefly, a 3–5 cm thoracic wall incision was made, with the visible marked line serving to guide PN resection. A wedge resection approach was performed when possible, with segmental resection instead being performed in cases where VATS visualization was not sufficient to guarantee adequate surgical margins. The resected pulmonary parenchymal tissue samples were then sent for rapid pathological assessment in the Department of Pathology. Lobectomy and systematic lymph node dissection were performed for patients diagnosed with invasive lung tumors, while other patients did not undergo additional resective procedures.

### Endpoints and study definitions

The technical success of the SCN localization procedure was the primary endpoint for this study, while secondary endpoints included the duration of localization, localization-associated complications, the technical success of VATS wedge/segmental resection, VATS duration, intraoperative blood loss, and final patient diagnoses.

SCN localization was considered to be a technical success when the marked line could be visualized and dislodgement did not occur [9]. Wedge/segmental resection was considered successful when the target PN was present within the resected parenchymal tissue [9]. Pulmonary hemorrhage was detected when CT scans revealed new-onset consolidative or ground-glass opacity located near the needle path [13]. A visual analog scale (VAS) was used to assess patient pain levels, with scores ranging from 0 (no pain) to 10 (worst possible pain) [14].

## Results

### Patient characteristics

The 12 patients enrolled in this study each harbored a single PN located behind the scapula (Table 1). These patients included 4 males and 8 females, with a median age of 57 years (range: 39–73).

**Table 1:** Baseline data of the 12 patients

	Age	Gender	BMI	Smoking	Tumor history	Emphysema	Location	Nature	Diameter (mm)	Lesion-pleura distance (mm)
1	53	Female	23.6	No	No	No	Right upper	GGN	7	6
2	55	Female	22.5	No	No	No	Right upper	GGN	8	1
3	57	Female	25.2	No	No	No	Left upper	Solid	6	12
4	58	Female	23.2	No	No	No	Right lower	GGN	7	9
5	57	Male	24.8	No	No	No	Right upper	GGN	10	15
6	47	Female	21.6	No	No	No	Left upper	GGN	7	12
7	66	Female	24.1	No	No	No	Right upper	GGN	9	11
8	64	Male	25.5	Yes	No	Yes	Left upper	GGN	7	6
9	73	Male	25.4	No	No	No	Right upper	Solid	7	8
10	46	Female	22.7	No	No	No	Right upper	GGN	8	7
11	39	Female	21.2	No	No	No	Right upper	GGN	9	2
12	66	Male	23.3	Yes	No	Yes	Right upper	GGN	8	1

BMI: body-mass index; GGN: ground glass nodule

### Pulmonary nodule characteristics

Lesions identified in study participants included 10 GGNs and 2 solid PNs with a median 8 mm diameter (range: 6–10 mm) and a median PN-pleura distance of 8 mm (range: 1–15 mm). Of these PNs, 8, 3, and 1 were respectively located in the right upper lobe, left upper lobe, and right lower lobe.

### CT-guided SCN localization

Technical success was achieved for the CT-guided transscapular SCN localization procedure in all patients without any complications proximal to the scapula (Table 2). The median duration of the localization procedure was 12 min (range: 7–20 min), and the median post-localization VAS score was 3 (range: 1–4). Of these patients, 2 (16.7%) and 1 (8.3%) respectively experienced post-localization pneumothorax and pulmonary hemorrhage, none of which resulted in the delay of the VATS procedure.

**Table 2:** Details of the CT-guided localization

	Technical success	Patients’ position	Length of the SCN (mm)	Localization time (min)	Post-localization VAS score	Complication
1	Yes	Prone	100	9	3	Pneumothorax
2	Yes	Prone	100	18	4	None
3	Yes	Prone	150	13	2	None
4	Yes	Prone	100	7	3	None
5	Yes	Prone	150	20	3	None
6	Yes	Prone	100	9	3	Pulmonary hemorrhage
7	Yes	Prone	150	14	2	None
8	Yes	Prone	100	7	3	None
9	Yes	Prone	100	10	2	None
10	Yes	Prone	100	11	2	None
11	Yes	Prone	100	15	3	None
12	Yes	Prone	100	18	1	Pneumothorax

SCN: Sens-cure needle; VAS: visual analogue scale

### VATS

Technical success was achieved for VATS wedge resection procedures in all patients, with no patients having undergone segmental resection (Table 3). Rapid pathological assessment of these lesions revealed 3 benign lesions, 5 mini-invasive adenocarcinomas, and 4 invasive adenocarcinomas. These latter 4 patients thus underwent subsequent lobectomy with systematic lymph node dissection. The median VATS procedure duration was 80 min (range: 45–250 min), and the median blood loss was 10 mL (range: 10–100 mL). All final diagnoses in this patient cohort were identical to the results of rapid pathological testing.

**Table 3:** Details of the VATS procedures

	Technical success of WR	Types of surgery	VATS time (min)	Blood loss (ml)	Diagnoses
1	Yes	WR alone	80	10	MIA
2	Yes	WR + lobectomy	140	100	IA
3	Yes	WR alone	70	50	Benign
4	Yes	WR alone	80	100	Benign
5	Yes	WR + lobectomy	180	50	IA
6	Yes	WR alone	60	10	MIA
7	Yes	WR alone	70	10	MIA
8	Yes	WR + lobectomy	250	100	IA
9	Yes	WR alone	45	10	Benign
10	Yes	WR alone	80	10	MIA
11	Yes	WR alone	50	10	MIA
12	Yes	WR + lobectomy	160	10	IA

VATS: video-assisted thoracoscopic surgery; WR: wedge resection; MIA: mini-invasive adenocarcinoma; IA: invasive adenocarcinoma

## Discussion

Preoperative CT-guided localization strategies are widely used in patients with PNs prior to VATS-mediated sublobar resection procedures in order to reduce the need for VATS-based anatomic resection or thoracotomy [15]. The present results highlight the safety, feasibility, and efficacy of CT-guided SCN localization for PNs located behind the scapula. When bony structures obstruct the needle pathway, alternative pathways must generally be selected to facilitate effective localization [16]. However, these alternative pathways inevitably result in a longer intra-pulmonary needle pathway, potentially increasing procedure-related complication rates [17] while also increasing the volume of resected lung parenchymal tissue [18].

Scapular puncture is the key step in this localization procedure. The 100% technical success rate achieved through the transscapular approach in this study may be attributable to the fact that the scapula is fairly thin, and that the needle was rotated as it was advanced following scapular puncture. This transscapular SCN localization strategy can also minimize the volume of resected lung parenchyma.

Relative to coil localization strategies, SCN localization can lower the operative duration while maintaining a similar risk of complications [10]. The median localization time in this study (12 min) was slightly longer than the 9.4–10.6 min reported previously in studies of the CT-guided SCN localization for PNs [10, 19], potentially owing to the fact that scapular puncture was performed prior to localization.

The design of the SCN was based upon traditional hook-wire characteristics [9]. However, in contrast to traditional hook-wire localization in which a piece of stainless steel remains visible protruding from the body once localization is complete, the soft, flexible sutures connected to the SCN anchor claw can be pushed into the pleural cavity when localization is complete, thereby potentially avoiding tension on the anchor claw resulting from breathing-related movement of changes in patient positioning, lowering associated risks of dislodgement or procedure-related complications [9].

All patients in this study population underwent successful VATS wedge resection procedures. This high success rate was largely attributable to the success of localization in all cases. The tri-colored marking sutures also allowed for a more effective assessment of PN depth and the area to be resected during the VATS procedure [9].

This study is subject to some limitations. As these analyses were retrospective in design, they are susceptible to potential bias. This was a single-center study, and the reproducibility of results in centers that are not experienced in the use of this technique is uncertain. In addition, as PNs located beneath the scapula are relatively rare, the sample size in this study was limited, and defining an appropriate control group was challenging. As such, drawing definitive conclusions based on these results is difficult, and further in-depth follow-up research is warranted.

## Conclusion

In summary, preoperative CT-guided transscapular SCN localization represents a simple, safe, and effective means of localizing PNs positioned behind the scapula.

## Data Availability

The data that support the findings of this study are available from the corresponding author upon reasonable request.

## Abbreviations

CT: computed tomography
GGN: ground-glass nodule
IA: invasive adenocarcinoma
MIA: mini-invasive adenocarcinoma
PN: pulmonary nodule
SCN: Sens-cure needle
VATS: video-assisted thoracoscopic surgery
